# Relationship between acupuncture and transient receptor potential vanilloid: Current and future directions

**DOI:** 10.3389/fnmol.2022.817738

**Published:** 2022-11-03

**Authors:** Dan Luo, Li Liu, Hai-ming Zhang, Yu-dian Zhou, Min-feng Zhou, Jin-xiao Li, Zhao-min Yu, Rui Chen, Feng-xia Liang

**Affiliations:** ^1^Department of Acupuncture and Moxibustion, Hubei University of Traditional Chinese Medicine, Wuhan, China; ^2^Department of Respiratory, Wuhan No. 1 Hospital, Wuhan, China; ^3^Department of Pathology, Wuhan No. 1 Hospital, Wuhan, China; ^4^Department of Oncology, Integrated Traditional Chinese and Western Medicine, The Central Hospital of Wuhan, Tongji Medical College, Huazhong University of Science and Technology, Wuhan, China; ^5^Department of Integrated Traditional Chinese and Western Medicine, Union Hospital, Tongji Medical College, Huazhong University of Science and Technology, Wuhan, China; ^6^Department of Oncology, Hubei Province Hospital of Integrated Traditional Chinese and Western Medicine, Wuhan, China

**Keywords:** acupuncture, TRPV channels, autonomic nervous system, central nervous system, peripheral sensory nervous

## Abstract

Acupuncture is a common complementary and alternative therapy around the world, but its mechanism remains still unclear. In the past decade, some studies indicated that transient receptor potential vanilloid (TRPV) channels play a great role in the response of acupuncture stimulation. In this article, we discussed the relationship between acupuncture and TRPV channels. Different from inhibitors and agonists, the regulation of acupuncture on TRPV channels is multi-targeted and biphasic control. Acupuncture stimulation shows significant modulation on TRPV1 and TRPV4 at the autonomic nervous system (ANS) including central and peripheral nervous systems. On the contrary, the abundant expression and functional participation of TRPV1 and TRPV4 were specific to acupuncture stimulation at acupoints. The enhancement or inhibition of TRPV channels at different anatomical levels will affect the therapeutic effect of acupuncture. In conclusion, TRPV channels help to understand the principle of acupuncture stimulation, and acupuncture also provides a potential approach to TRPV-related trials.

## Introduction

Acupuncture originated in China over 3,000 years ago. In acupuncture theory, stimulation at certain areas, also known as acupoints, with needles could dynamically harmonize Yin-Yang and Qi to cure diseases (Kaptchuk, 2002). Nowadays, acupuncture is confined as an important complementary and alternative therapy all over the world (Rubin, 2019; Slomski, 2019). Some systematic reviews and meta-analysis have confirmed that acupuncture has encouraging effects on pain (He et al., 2020) and obesity (Kim et al., 2018).

Neurobiological mechanisms underlying acupuncture’s effectiveness have been widely discussed in modern research. Acupuncture is effective on the endocannabinoid system (Hu et al., 2017), purinergic signaling (Tang et al., 2016), and neuro-immune microenvironment (Gong et al., 2020). Current studies have shown that acupuncture plays a great role in the autonomic nervous system (ANS) (Shu et al., 2016). Liu et al. (2021) performed studies on acupuncture relieving inflammation, which showed that both vagal-adrenal axis and NPY-expressing sympathetic pathway (Liu et al., 2020) could be regulated by acupuncture stimulation at certain acupoints through specific autonomic pathways. It is worth mentioning that either anti-inflammatory or pro-inflammatory effects of acupuncture depend on the state of diseases. Although several biological correlates in ANS may explain the principle of acupuncture (Lim et al., 2016), the biological basis of stimulation at acupoints affecting physiology and pathology of internal organs remains unknown.

Since transient receptor potential vanilloid (TRPV) 1, which is sensitive to both heat and capsaicin, was first found in neurons in 1997 (Caterina et al., 1997), other five TRPV family members including TRPV2-6 have been identified in the next few years (Smith et al., 2002; Patapoutian et al., 2003; van de Graaf et al., 2003). Recent studies showed that TRPV channels are widely expressed in various excitable and non-excitable cell types in the human body. TRPV channels not only respond to thermosensation (Paricio-Montesinos et al., 2020) but also play a great role in the physiological or pathological processes of pain, inflammation, immunity, diabetes, and obesity (Jordt and Ehrlich, 2007; Samanta et al., 2018).

Physical stimulation such as pressure, vibration, pain, and temperature can be felt by sensory receptors at the peripheral nervous system, and the signal will be further passed to the central nervous system (CNS) through dorsal root ganglia (DRG). The operation of acupuncture produces pain, pressure, and vibration at the acupoints for neurobiological regulations. Hence, it is reasonable to assume that there is a specific relationship between acupuncture and TRPV channels. To the best of our knowledge, some studies have shown that acupuncture is closely associated with TRPV1 and TRPV4, which is elaborated in this review.

## Transient receptor potential vanilloid channels

As sensory receptors, TRPV channels are sensitive to various tissue-damaging signals and have a strong link with signaling pathways such as phosphorylation of protein kinase A (PKA) and protein kinase C (PKC) (Por et al., 2013). Hence, TRPV channels are also considered as nociceptors (Satheesh et al., 2016). TRPV channels in the CNS play a great role not only in pain but also in neuropsychiatric disorders, such as depression, stress, and anxiety (Singh et al., 2019). It was found that all kinds of tissues and organs with TRPV channels expression contribute to a plethora of physiological or pathophysiological effects (Seebohm and Schreiber, 2021).

TRPV1 has been the most widely studied TRPV channel in the past decade. TRPV1 expresses in both neuronal cells, such as peripheral sensory neurons (C- and Aδ-fibers), DRG, trigeminal ganglia, and vagal ganglia (Cao et al., 2013), and non-neuronal cells, such as skin and muscles (Cavanaugh et al., 2011). After TRPV1 was knocked out, mice exhibited no vanilloid-evoked pain behavior and impaired nociception shown in inflammation pain mice models (Caterina et al., 2000). But it should be noted that TRPV1 agonists are also effective on neuropathic pain. Agonists could over-activate TRPV1 channels and lead to internalization and subsequent desensitization of afferent nerve endings or degeneration of neurons by Ca^2+^-induced neurotoxicity (Fischer et al., 2020).

Capsaicin, an important component in spiciness with a spicy sensation, could selectively block pain signals at primary afferent neurons targeting TRPV1 (Szolcsanyi, 2008). Besides capsaicin, TRPV1 could be activated by many physical and chemical stimuli, and activation pathways exist for specific stimuli (Yang and Zheng, 2017). The extracellular pore domain and transmembrane domains of TRPV1 could respond to different stimulation from physical and chemical inputs (Zheng, 2013). Many small molecules as potential analgesics aimed to inhibit TRPV1, but most of them failed in clinical trials due to severe side effects (Carnevale and Rohacs, 2016). Until now, only 8% capsaicin patch was approved by the European Union and the US Food and Drug Administration (FDA) for the treatment of postherpetic neuralgia, namely, peripheral neuropathic pain (Basith et al., 2016).

Similar to TRPV1, TRPV4 channels are broadly expressed in organs and tissues to participate in many physiological and pathophysiological processes. The therapeutic effect of TRPV4 antagonism on pain, gastrointestinal disorders, and respiratory diseases has been suggested through animal studies (Grace et al., 2017). GSK2798745, a potent and selective TRPV4 inhibitor, has been investigated in early phase clinical trials for heart failure (Brooks et al., 2019). To date, TRPV4 agonists could lead to unpredictable toxicity during systemic activation of TRPV4 and only suggested local delivery (Lawhorn et al., 2020).

## Acupuncture stimulation

Acupuncture is a treatment that involves inserting needles at specific acupoints in the body. In traditional Chinese medicine theory, the key to the curative effect of acupuncture treatment lies in the human body’s response to acupuncture stimulation, including heaviness, numbness, soreness, and distension, which is also called “De Qi” (Mao et al., 2007). It is believed that acupuncture stimulation could activate mechanically sensitive pain fibers and various types of lesser-known deep tissue receptors (Zhao, 2008).

The analgesic effect of acupuncture has been studied for a long time. Brain imaging shows that acupuncture could alter activation patterns in brain areas against pain processing (Huang et al., 2012). Endorphins (Han, 2004), serotonin (Zhang Y. et al., 2012), and various neuromodulators and neurotransmitters in CNS and/or peripheral nervous system (PNS) can be regulated by acupuncture stimulation. A systematic review based on quantitative sensory testing (QST) has found that acupuncture significantly changed the sensory threshold and activated neuromodulation (Baeumler et al., 2014). A large number of preclinical trials have shown that the analgesic effect of electroacupuncture is optimistic, and the peripheral, spinal, and supraspinal mechanisms related to the activation of a variety of bioactive chemicals show more difference in health than that in pain conditions (Zhang et al., 2014).

## Acupuncture affects transient receptor potential vanilloid channels

**FIGURE 1:**
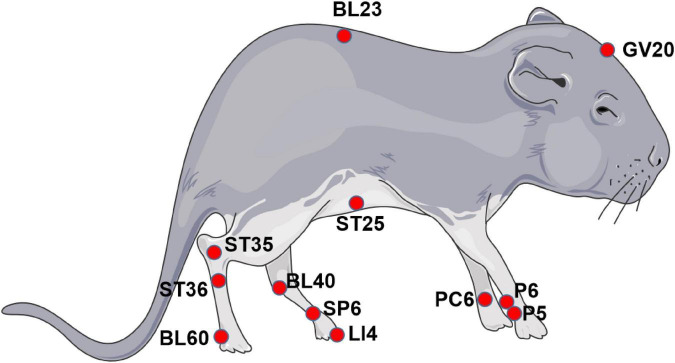
Acupoints in murine. Acupuncture delivery is through the insertion of needles into the muscle at acupoints with 1–3 mm depth and triggering of a local reaction by means of manual manipulation or electrical stimulation. Sham acupuncture is usually performed in the tissue adjacent to the targeted acupoint without manual operation or electrode connection.

A total of 24 animal studies were included in this article, and acupuncture/electroacupuncture showed significantly beneficial effects. The regulation of acupuncture/electroacupuncture to TRPV channels differs in models (Table 1). To help readers understand the implementation methods of acupuncture, we listed the acupoints of the animal related to this article in Figure 1 (Jin et al., 2018).

**TABLE 1:** Acupuncture affects TRPV channels.

Models	Acupuncture delivery	Acupoints	Target organs	Main results	References
Mice, Inflammatory pain models	MA	ST36	Muscle at ST36	TRPV1 and TRPV4 channels were abundantly expressed	Wu et al., 2014
			epimysium at ST36		
			Subcutaneous loose connective tissue at ST36		
			Neural tissue at ST36		
Mice, Inflammatory pain models	EA, 2 Hz 1 mA	ST36	DRG	TRPV1 channel overexpression was decreased	Liao et al., 2017
			SCDH		
Mice, Inflammatory pain models	EA, 2 Hz 1 mA	ST36	DRG	TRPV1 channel overexpression was decreased	Yang et al., 2017
			Spinal cord		
Mice, Inflammatory pain models	EA, 2 Hz 1 mA	LI4	Prefrontal cortex	TRPV1 channel overexpression was decreased	Yen et al., 2019
			Hypothalamus		
			Periaqueductal gray	TRPV1 channel suppression was reversed	
Mice, Inflammatory pain models	EA, 2 Hz 1 mA	ST36	Cerebellum lobules V, VIa and VII	TRPV1 channel overexpression was decreased	Inprasit and Lin, 2020
Mice, Chronic pain and depression models	EA, 2 Hz 1 mA	ST36	Cerebellum lobules VI, VII, VIII	TRPV1 channel inhibition was revised	Lottering and Lin, 2021
Rats, Inflammatory pain models	EA, 2 Hz, 100 Hz, 2/100 Hz 0.5-1.0-1.5 mA	ST36	L4-6 DRG neurons	TRPV1 channel overexpression was decreased high-frequency EA was more effective	Fang et al., 2018
Mice, Fibromyalgia models	EA, 2 Hz 1 mA	ST36	DRG neurons	TRPV1 channel overexpression was decreased	Lin et al., 2015
			Spinal cord		
				TRPV4 channel overexpression was decreased	
Mice, Fibromyalgia models	EA, 2 Hz 1 mA	ST35	Thalamus	TRPV1 channel overexpression was decreased	Hsu et al., 2020
			Amygdala		
			Somatosensory cortex		
Rats, carcinoma cell inoculation to cancer pain models	EA, 2 Hz 1 mA	ST36	DRG neurons	TRPV1 channel overexpression was decreased	Zhang Z. et al., 2012
Mice, cold stress-induced nociception and depression models	EA, 2 Hz 1 mA	ST36	Medial prefrontal cortex	TRPV1 channel suppression was reversed	Lin et al., 2020
			Hippocampus	TRPV1 channel overexpression was decreased	
			Periaqueductal gray		
			Amygdala		
Rats, high fat diet-induced obese models	EA, 10 Hz 1 mA	ST36	Medulla regions	TRPV1 channel suppression was reversed	Ji et al., 2013
			Skin at ST36		
Rats, paclitaxel-induced peripheral neuropathy models	EA, 2 Hz 0.5–1.5 mA	ST36 and BL60	L4-6 DRG neurons	TRPV1 channel overexpression was decreased	Li et al., 2019
Rats, MCAo models	EA, 2 Hz 2 mA	GV20	Hippocampal CA1 areas	TRPV1 channel overexpression was decreased	Lin and Hsieh, 2010
Rats, MCAo models	EA, 2/100 Hz 2 mA	GV20, BL23 and, SP6	Hippocampal	TRPV1 channel overexpression was decreased	Long et al., 2019
Mice	EA, 2 Hz 1 mA	ST36	DRG neurons	TRPV1 channel was upregulated	Choowanthanapakorn et al., 2015
			Spinal cord		
Mice, motion sickness models	EA, 2 Hz 1 mA	PC6	Thalamus	TRPV1 channel overexpression was decreased	Inprasit et al., 2018
			Hypothalamic		
			Brain stem		
Rats	EA, 1 mA	BL40	Subepidermal nerve fibers at BL40	TRPV1 channel was upregulated	Abraham et al., 2011
Mice	MA	ST36	Peripheral DRG neurons	Components of the TRPV1-related signaling pathway was upregulated	Chen et al., 2018
	EA 2, 15, 50 Hz and 1 mA		Somatosensory cortex		
Rats	EA 2, 15 Hz and 1 mA	ST36	Splenic CD4 + T cells	TRPV1 channel was upregulated	Chen et al., 2017
Rats, gastric distension to cardiovascular reflexes models	MA	P5 and P6	C7-8 DRG neurons	TRPV1 channel was upregulated	Guo et al., 2018
	EA, 2 Hz 0.3–0.5 mA				

MA, manual acupuncture; EA, electroacupuncture; MCAo, middle cerebral artery occlusion; DRG, dorsal root ganglion; SCDH, spinal cord dorsal horn.

The relationship between acupuncture and TRPV channels involves the whole PNS, including sensory receptors and afferent nerves. Acupuncture could significantly increase the subepidermal nerve fibers with high expression of TRPV1 (Abraham et al., 2011). DRG neurons are believed to act as the bridge for acupuncture stimulation projecting to CNS. However, the effect of acupuncture on TRPV channels at DRG may be completely opposite in different models. In studies focused on diseases related to pain, the overexpression of TRPV1 channel in DRG neurons by drugs or surgery, which causes pain, would be reversed by acupuncture (Zhang Z. et al., 2012; Lin et al., 2015; Liao et al., 2017; Yang et al., 2017). But in other studies based on normal animals or obese models, acupuncture enhanced the expression of TRPV1 channels in DRG neurons (Ji et al., 2013; Choowanthanapakorn et al., 2015; Chen et al., 2018). Acupuncture at different acupoints would lead to TRPV channel changes in different DRG neurons. Stimulation at ST36 and BL40 is likely passed to L4-6 DRG neurons (Li et al., 2019), while acupuncture at P5 and P6 targets at C7-8 DRG neurons (Guo et al., 2018). Meanwhile, the effect of electroacupuncture on regulating TRPV channels is positively correlated with frequency (Fang et al., 2018).

It is noticed that acupuncture stimulation could modulate TRPV channels in the brain and spinal cord (Liao et al., 2017; Yang et al., 2017; Inprasit et al., 2018). Similar to PNS, the regulation of acupuncture on CNS is also bidirectional. Acupuncture significantly inhibits the trend of TRPV channels’ overexpression in pain-related models but promotes TRPV channels’ expression in obesity (Ji et al., 2013) or normal condition (Choowanthanapakorn et al., 2015). Meanwhile, stimulation at acupoints could lead to a multi-targeted effect on the central nervous system. For example, after stimulation at ST36, the changes of TRPV channels in the spinal cord (Yang et al., 2017); cerebellum lobules V, VIa, VI, VII, and VIII (Inprasit and Lin, 2020; Lottering and Lin, 2021); hippocampus; periaqueductal gray; and medial prefrontal cortex (Lin et al., 2020) could be observed.

In addition to nerves, acupuncture stimulation could also directly regulate the expression of TRPV1 and TRPV4 channels in different anatomical layers of skin at acupoints including muscles, epimysium, and subcutaneous loose connective tissue (Ji et al., 2013; Wu et al., 2014). It is noticed that electroacupuncture at ST36 enhances immune cytokines by promoting the TRPV1 channels in splenic CD4^+^ T cells (Chen et al., 2017). Mast cells could also be activated by acupuncture through TRPV2 channels to release histamine (Huang et al., 2018).

## Transient receptor potential vanilloid channels influence the effect of acupuncture

The use of TRPV gene knockout, agonist, and antagonism provides us with an opportunity to understand the relationship between acupuncture and TRPV from another perspective (Table 2). When acupuncture enhances TRPV expression in wild models, the effect of acupuncture could be significantly inhibited after TRPV gene knockout (Yu et al., 2016; Huang et al., 2018). If TRPV over-expression is related to the progress of disease such as hyperpathia, the TRPV gene knockout mimics the analgesic effect of acupuncture (Liao et al., 2017; Yang et al., 2017). Delivery of TRPV agonist or antagonist on different areas of PNS could lead to different effects of acupuncture. The injection of TRPV1 antagonist into ST36 could mimic the acupuncture-like analgesic effect, but it was not replicated through the injection of TRPV4 agonist (Wu et al., 2014). The injection of TRPV1 antagonist into P5 and P6 could inhibit the modulation of sympathoexcitatory responses in manual acupuncture but not in electroacupuncture (Guo et al., 2018). A study performed by Fang et al. (2018) found that the injection of capsaicin into the dorsum of the foot could exhibit an analgesic effect similar to acupuncture. But Li et al. (2019) found that the injection of capsaicin into the dorsum of the foot reversed the effect of acupuncture and TRPV1 antagonist showed a contrary result.

**TABLE 2:** TRPV channels could influence the effect of acupuncture.

Models	Intervention on TRPV	Methods	Main results	References
Mice inflammatory pain models	TRPV1 agonist	Capsaicin injected into ST36	Replicated the acupuncture-like analgesic effect	Wu et al., 2014
	TRPV4 agonist	GSK1016790A injected into ST36	Did not induce an analgesic effect	
Mice inflammatory pain models	TRPV1 antagonist	TRPV1 gene knockout	Replicated the acupuncture-like analgesic effect	Liao et al., 2017
Mice inflammatory pain models	TRPV1 antagonist	TRPV1 gene knockout	Replicated the acupuncture-like analgesic effect	Yang et al., 2017
Mice inflammatory pain models	TRPV1 antagonist	TRPV1 gene knockout	Replicated the acupuncture-like analgesic effect	Yen et al., 2019
Mice, chronic pain and depression models	TRPV1 antagonist	TRPV1 gene knockout	There is no significant difference with the model group	Lottering and Lin, 2021
Mice inflammatory pain models	TRPV1 agonist	Capsaicin injected into the dorsum of the foot	Replicated the acupuncture-like analgesic effect	Fang et al., 2018
Mice, fibromyalgia models	TRPV1 antagonist	TRPV1 gene knockout	Replicated the acupuncture-like analgesic effect	Lin et al., 2015
Mice, cold stress-induced nociception and depression models	TRPV1 antagonist	TRPV1 gene knockout	Replicated the acupuncture-like analgesic effect	Lin et al., 2020
Rats, paclitaxel-induced peripheral neuropathy models	TRPV1 agonist	Capsaicin injected into dorsal part of the ipsilateral hind paw	Inhibited the analgesic effect of acupuncture	Li et al., 2019
	TRPV1 antagonist	AMG9810 injected into dorsal part of the ipsilateral hind paw	Replicated the analgesic effect of acupuncture	
Rats, MCAo models	TRPV1 agonist	Capsaicin, subcutaneous injection	Inhibited the analgesic effect of acupuncture	Long et al., 2019
	TRPV1 antagonist	AMG-517, intraperitoneal injection	Replicated the analgesic effect of acupuncture	
Mice	TRPV1 antagonist	TRPV1 gene knockout	Inhibited the weight-loss effect of acupuncture	Choowanthanapakorn et al., 2015
Mice, motion sickness models	TRPV1 antagonist	TRPV1 gene knockout	Replicated the acupuncture-like relieving motion sickness symptoms effect	Inprasit et al., 2018
Mice	TRPV1 antagonist	TRPV1 gene knockout	Inhibited the phosphorylated effect of acupuncture	Chen et al., 2018
Rats	TRPV1 antagonist	TRPV1 gene knockout	Inhibited the CD4 + T cells active effect of acupuncture	Chen et al., 2017
Rats, gastric distension to cardiovascular reflexes models	TRPV1 antagonist	SiRNA, injected into C7-8 DRG neurons	Inhibited the inhibition of reflex increases in blood pressure by MA but not in EA	Guo et al., 2018
		Iodoresiniferatoxin, injected into P5 and P6	Inhibited the modulation of sympathoexcitatory responses by MA but not in EA	
Mice	TRPV1 antagonist	TRPV1 gene knockout	Inhibited the analgesic effect of EA and significant in higher intensity	Xin et al., 2016
Rats, acute adjuvant arthritis models	TRPV2 antagonist	TRPV2 gene knockout	Inhibited the acupuncture activation effect of mast cells and analgesic effect	Huang et al., 2018
Rats	TRPV1 antagonist	TRPV1 gene knockout	Inhibited the effect of acupuncture in suppressing the motor activity of the jejunum in an intensity-dependent manner	Yu et al., 2016

## Acupuncture and transient receptor potential vanilloid channels participate in complex molecular networks

No matter how acupuncture regulates TRPV channels or how TRPV channels influence the effect of acupuncture as described above, TRPV channels mediate the communication between acupuncture and body tissues (Table 3). Through TRPV channels, acupuncture could regulate complex molecular networks, including adenosine triphosphate (ATP), extracellular signal-regulated kinase (ERK), toll-like receptor 4 (TLR4), and others. Phosphorylation of downstream molecules was widely found after acupuncture-regulated TRPV channels (Inprasit et al., 2018). Inprasit and Lin (2020) and Lottering and Lin (2021) performed a series of studies to clarify the role of acupuncture and TRPV in the cerebellum lobules. Both complete Freund’s adjuvant (CFA) and acid saline (AS) could lead to chronic pain, but the expression of TRPV1 in the cerebellum lobules is completely opposite. Although it is observed that the analgesic effect of acupuncture at ST36 is obvious in both studies, the regulation of TRPV1 and phosphorylation of molecules in MAPK pathways by acupuncture are quite different. Behavioral results indicate that the mechanism of TRPV1 and related inflammatory factors in pain is not single and decisive in different models. The AS model restores the general concept of hyperalgesia caused by the pathological overexpression of TRPV and inflammation, and the influence of TRPV1 and inflammatory factors on pain sensation was not significant in the CFA model. In addition, the low expression of TRPV1 and inflammatory factors also leads to the formation of depression in mice after the injection of CFA.

**TABLE 3:** Acupuncture and TRPV channels play a great role in related molecules and pathways.

Models	Acupuncture delivery	Acupoints	TRPV	Targets	Related molecules or pathways	References
Mice inflammatory pain models	MA	ST36	TRPV1 and TRPV4	Cell membrane at muscle, epimysium, and neuron	Promote ATP signaling	Wu et al., 2014
Mice inflammatory pain models	EA, 2 Hz 1 mA	ST36	TRPV1	DRG and SCDH	Inhibit PI3K, AKT, CREB, NF-κB, Nav1.7, and Nav1.8	Liao et al., 2017
Mice inflammatory pain models	EA, 2 Hz 1 mA	ST36	TRPV1	DRG and Spinal cord	Inhibit pPKA, pPI3K, pPKC, pERK, pp38, pJNK, pCREB, pNF-κB, Nav1.7, Nav1.8, GFAP, S100B, and RAGE	Yang et al., 2017
Mice inflammatory pain models	EA, 2 Hz 1 mA	LI4	TRPV1	Brain	Inhibit pPKA, pPI3K, pPKC, pERK, pp38, pJNK, pCREB, pNF-κB, Nav1.7, Nav1.8	Yen et al., 2019
Mice inflammatory pain models	EA, 2 Hz 1 mA	ST36	TRPV1	Cerebellum lobules V, VIa and VII	Inhibit pPI3K, pmTOR, pAkT, pERK, pPKCε, pPKAIIα, pNFkB, pCREB, and S100B	Inprasit and Lin, 2020
Mice, chronic pain and depression models	EA, 2 Hz 1 mA	ST36	TRPV1	Cerebellum lobules VI, VII, VIII	Promote pmTOR, pPI3K, NMDAR1, pPKCε, pAkt, TrkB, pNFκB, GABAAα1, pPKAIIα, pCREB, and Perk	Lottering and Lin, 2021
Mice fibromyalgia models	EA, 2 Hz 1 mA	ST36	TRPV1 and TRPV4	L5 DRG neurons	Inhibit pERK signaling	Lin et al., 2015
Mice fibromyalgia models	EA, 2 Hz 1 mA	ST35	TRPV1	Brain	inhibit pERK signaling	Hsu et al., 2020
Mice, cold stress-induced nociception and depression models	EA, 2 Hz 1 mA	ST36	TRPV1	Medial prefrontal cortex, hippocampus and periaqueductal gray	Promote pPKA, pPI3K, pPKC, pAKT, pmTOR, pERK, pp38, pJNK, pCREB, and pNFκB	Lin et al., 2020
Rats, high fat diet-induced obese models	EA, 10 Hz 1 mA	ST36	TRPV1	Nucleus tractus solitarius/gracile nucleus regions	Promote nNOS	Ji et al., 2013
				Skin at ST36		
Rats, paclitaxel-induced peripheral neuropathy models	EA, 2 Hz 0.5 to 1.5 mA	ST36 and BL60	TRPV1	L4-6 DRG neurons	Inhibit TLR4 and MyD88 signaling	Li et al., 2019
Rats, MCAo models	EA, 2/100 Hz 2 mA	GV20, BL23 and, SP6	TRPV1	Hippocampal	Inhibit pp38	Long et al., 2019
Mice	EA, 2 Hz 1 mA	ST36	TRPV1	DRG and spinal cord	Promote pPKA, pPKC, and pERK signaling	Choowanthanapakorn et al., 2015
Mice, motion sickness models	EA, 2 Hz 1 mA	PC6	TRPV1	Thalamus	Inhibit pPI3K, pAKT, pmTOR, pERK, pp38, npJNK, pCREB, and pNFκB	Inprasit et al., 2018
Rats	EA, 1 mA	BL40	TRPV1	Subepidermal nerve fibers, C-fibers and A-δ fibers	Promote nNOS	Abraham et al., 2011
Mice	MA and EA 2, 15, 50 Hz and 1 mA	ST36	TRPV1	DRG and somatosensory cortex	Promote ppPKA, pPI3K, pPKC-pERK, pAKT and pNR1-pCaMKII pathway,	Chen et al., 2018
Rats	EA 2, 15 Hz and 1 mA	ST36	TRPV1	Splenic CD4 + T cells	Promote Ca2 + signaling	Chen et al., 2017
Rats, gastric distension to cardiovascular reflexes models	MA	P5 and P6	TRPV1	C7-8 DRG neurons	Promote pERK signaling	Guo et al., 2018
	EA, 2 Hz 0.3-0.5 mA			Group III and IV bimodal sensory afferent nerves		
Rats acute adjuvant arthritis models	MA	ST36	TRPV2	Mast cells	Active histamine H1 and adenosine A1 receptor	Huang et al., 2018
Rats	EA, 2 to 15 Hz	ST25	TRPV1	-	Promote sympathetic pathway	Yu et al., 2016

ATP, adenosine triphosphate; DRG, dorsal root ganglion; pERK, phosphoactivation of extracellular signal-regulated kinase; pPKA, phosphorylated protein kinase A; pCREB, cAMP-response-element-binding protein; NTS, nucleus tractus solitarius; nNOS, neuronal nitric oxide synthase; TLR4, toll-like receptor 4; MyD88, myeloid differentiation primary response 88.

## Conclusion and future directions

**FIGURE 2:**
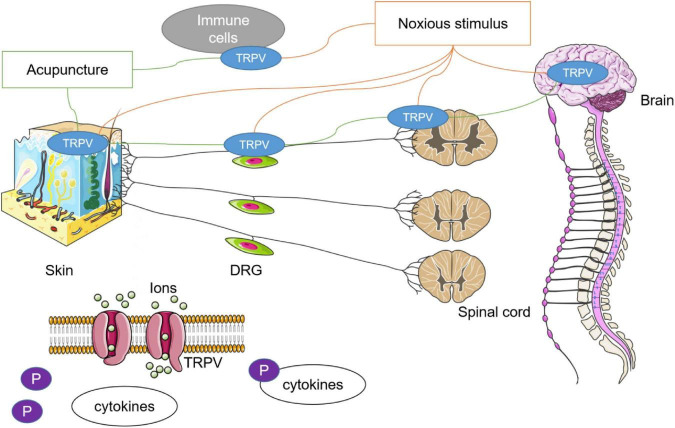
Relationship between acupuncture and TRPV channels. Noxious stimulus could lead to the imbalance of TRPV channel in multiple tissues. In many studies, it has been found that the therapeutic effect of acupuncture may be related to the agitation or antagonism of TRPV1 and TRPV4. The role of acupuncture in achieving systemic conditions through stimulation of specific acupoints may be closely dependent on the autonomic nervous system. Phosphorylation is an important mechanism in which acupuncture regulates downstream cytokines through TRPV channels.

Current studies have identified that there is a special relationship between acupuncture and TRPV channels including TRPV1 and TRPV4. First, stimulation at local acupoints can lead to systemic changes in TRPV channels, and the regulation of acupuncture to TRPV varies in different diseases or noxious stimuli. Second, the abundant expression and functional participation of TRPV1 and TRPV4 were specific to acupoints, and the enhancement or inhibition of TRPV channels at different anatomical levels will affect the therapeutic effect of acupuncture. Third, acupuncture and TRPV channels participate in complex molecular networks and that may explain the mechanism of acupuncture. All of the concepts are presented in Figure 2.

The regulation of acupuncture to TRPV channels is significantly different from that of TRPV agonist or antagonist. Similar to the balance of Yin and Yang pursued in acupuncture theory, acupuncture exhibits a bidirectional regulation to TRPV channels in multiple targets and contributes to an overall improvement of clinical symptoms and physiological functions in different diseases or stages of illness. In the past decades, TRPV1-targeted drugs have been long studied for human pain conditions (Iftinca et al., 2021). Many drugs attempting to block TRPV1 may lead to mixed results (Basso and Altier, 2017). Only the efficacy of topical use of TRPV agonists such as capsaicin patches has been recognized, but the systemic administration was not suggested because of the adverse effects on blood pressure, breathing, and other reflex pathways (Lu et al., 2020). The regulation of acupuncture to the p38 signaling pathway *via* TRPV in several nervous system diseases has been discussed by Wei and Hsieh (2020). Similar to this article, the role of acupuncture to p38 signal pathways is bidirectional, but it can improve the symptoms of a disease.

TRPV channels may also help us understand the principle of acupuncture. TRPV channels tend to be highly expressed at acupoints after acupuncture stimulation. Compared with sham acupuncture, only acupuncture stimulation at acupoints could cause TRPV response. The expression of TRPV and the effect of using TRPV agonist or antagonist were also different from various acupuncture methods. For example, TRPV is sensitive to the intensity and duration of electroacupuncture, which suggests that there might be a more precise adjustment between acupuncture and TRPV channels.

There are still some limitations. All studies in this review were based on animal models. Therefore, the results in humans are inconclusive. In a single study, the regulatory relationship between acupuncture and TRPV was clear, but these effects became complex after consideration of similar studies. This prevents us from simply defining acupuncture as an agonist or inhibitor, as capsaicin does. At the same time, only a few articles compared the changes of acupuncture efficacy after local use of TRPV-related drugs, making it difficult to evaluate acupuncture and existing TRPV agonists or antagonist. As described earlier, activation of TRPV is structurally specific and selective. However, there are no studies on the changes of TRPV structure after acupuncture intervention. With the cryo-EM resolution revolution, the structural insights into the gating mechanisms of TRPV channels developed rapidly (Pumroy et al., 2020), and the role of TRPV channels in the endoplasmic reticulum has also been noticed (Haustrate et al., 2020). Compared with the current TRPV-related drugs, the effectiveness and safety of acupuncture in pain have been widely discussed (Cherkin et al., 2003; Manheimer et al., 2005). In conclusion, acupuncture has a strong relationship with TPPV1 and TRPV4. Acupuncture may provide a viable intervention target to TRPV channels. TRPV channels also help us understand how acupuncture works, especially for pain-related diseases. But the mechanism is unclear between acupuncture and TRPV channels, and further study is still needed.

## Author contributions

DL: conceptualization. DL, LL, H-mZ, Y-dZ, M-fZ, J-xL, and Z-mY: data curation. DL, LL, and H-mZ: writing—original draft preparation. F-xL and RC: writing—review and editing. All authors read and agreed to the published version of the manuscript and contributed to the article and approved the submitted version.

## Abbreviations

TRPV: transient receptor potential vanilloid
ANS: autonomic nervous system
CNS: central nervous system
PNS: peripheral nervous system
MA: manual acupuncture
EA: electroacupuncture
MCAO: middle cerebral artery occlusion
DRG: dorsal root ganglion
SCDH: spinal cord dorsal horn
ATP: adenosine triphosphate
DRG: dorsal root ganglion
Perk: phosphoactivation of extracellular signal-regulated kinase
pPKA: phosphorylated protein kinase A
pCREB: cAMP-response-element-binding protein
NTS: nucleus tractus solitarius
nNOS: neuronal nitric oxide synthase
TLR4: toll-like receptor 4
MyD88: myeloid differentiation primary response 88.
